# The Combined Regulation of Long Non-coding RNA and RNA-Binding Proteins in Atherosclerosis

**DOI:** 10.3389/fcvm.2021.731958

**Published:** 2021-11-02

**Authors:** Yuanyuan Ding, Ruihua Yin, Shuai Zhang, Qi Xiao, Hongqin Zhao, Xudong Pan, Xiaoyan Zhu

**Affiliations:** ^1^Department of Neurology, The Affiliated Hospital of Qingdao University, Qingdao, China; ^2^Department of Critical Care Medicine, The Affiliated Hospital of Qingdao University, Qingdao, China

**Keywords:** long non-coding RNA, RNA-binding protein, atherosclerosis, endothelial cells, macrophages, smooth muscle cells

## Abstract

Atherosclerosis is a complex disease closely related to the function of endothelial cells (ECs), monocytes/macrophages, and vascular smooth muscle cells (VSMCs). Despite a good understanding of the pathogenesis of atherosclerosis, the underlying molecular mechanisms are still only poorly understood. Therefore, atherosclerosis continues to be an important clinical issue worthy of further research. Recent evidence has shown that long non-coding RNAs (lncRNAs) and RNA-binding proteins (RBPs) can serve as important regulators of cellular function in atherosclerosis. Besides, several studies have shown that lncRNAs are partly dependent on the specific interaction with RBPs to exert their function. This review summarizes the important contributions of lncRNAs and RBPs in atherosclerosis and provides novel and comprehensible interaction models of lncRNAs and RBPs.

## Introduction

Atherosclerosis is the main cause of large-artery atherosclerotic (LAA) stroke (1). While its etiology is complicated and multifactorial; the exact mechanism is still unknown. Generally, when stimulated by dyslipidemia, hypertension, or pro-inflammatory mediators, endothelial cells (ECs) are injured, which enhances the expression of cell adhesion molecules (AMs), causing leukocytes to adhere on their surface (2). Low-density lipoprotein (LDL) penetrates the ECs and the space between the ECs (3). Monocytes migrate and differentiate into tissue macrophages and can form macrophage-derived foam cells by endocytosing the oxidized modified LDL (ox-LDL), leading to intracellular cholesterol accumulation (2). Vascular smooth muscle cells (VSMCs) migrate into the intima and engulf lipids to form muscle-derived foam cells. Once the initial process is completed, the atherosclerotic plaque progresses owing to the persistent accumulation of lipids and foam cells. Atherosclerosis is usually asymptomatic; however, unstable plaques may rupture and provoke thrombosis. Therefore, addressing the molecular mechanism of atherosclerosis is crucial to lay the foundation and highlight the prevention and treatment of stroke.

Recent, research has identified a functional genetic material called long non-coding RNA (lncRNA), which exert significant biological roles in multiple diseases. Although, the function of lncRNA is complex and still controversial, there is sufficient evidence to suggest that many lncRNAs have important cellular functions (4). Significantly, lncRNA regulates plaque development in all stages. They are involved in the process of atherosclerosis such as the regulation of ECs, macrophages, and VSMCs (5). Yan et al. found that lncRNA-RNCR3 was significantly upregulated in ECs and VSMCs cultured *in vitro* after ox-LDL treatment, and downregulation of RNCR3 accelerated the progress of atherosclerosis, exacerbated hypercholesterolemia and inflammatory factor release, and decreased ECs and VSMCs proliferation (6). Furthermore, studies found that some lncRNAs may be regarded as novel diagnostic biomarkers in LAA (7). Some small-molecule epigenetics drugs have not only been approved by the US Food and Drug Administration but also shown effects in preclinical studies of atherosclerosis (8). However, despite studies have shown that lncRNAs have a variety of important functions, their mechanism of action by which they regulate atherosclerosis is still complex and poorly understood.

Several studies have supported that lncRNAs perform many different functions by directly interacting with RNA-binding proteins (RBPs) (9). In the cytoplasm, for instance, exert their function by interacting with RBP through sequence motifs or by forming unique structural motifs (4). The expression of most RBPs is ubiquitous, usually higher than the average level of cellular protein. With the increase in the number of RBPs in higher eukaryotes, the relative sizes of different RBPs against different RNA targets remain unchanged throughout the phylogeny. However, the non-codingRNA-binding protein family and mRBP family have the lowest conservation rates, only 20% of the ncRNA-binding protein family has a homologous family in yeast (10). Furthermore, it has been found that 98% of RBP homologous families are universally expressed, and their deep evolutionary protection supports their superior basic cell functions. Among them, 20% families have tissue-specific and pervasive analogs, and members of the family are enriched in certain tissues (10). RBPs bound to the same type of RNA usually affect the same tissue and exhibits similar pathology (10). Nevertheless, their molecular function is largely determined by the localization of lncRNA and RBPs (11). Given that lncRNAs represent pivotal regulators in atherosclerosis, it is not unexpected that RBPs play key roles in atherosclerosis. Similarly, it has previously been observed that the RBP human antigen R(HuR) can regulate the progress of atherosclerosis (12). Hence, we also intend to focus on the effect of RBPs for a better understanding of the molecular mechanism in atherosclerosis.

lncRNAs and RBPs are a major area of interest within the field of atherosclerosis. At present, the mechanism of lncRNAs and lncRNAs-RBPs interaction in atherosclerosis remains an area of high research interest. Therefore, it is important to elucidate these molecular interactions to better understand the underlying mechanisms of atherosclerosis. In this review, we first briefly introduce the biology of lncRNA and RBPs. Next, we discuss how lncRNAs and RBPs regulate endothelial cells, macrophages, and vascular smooth muscle cells in atherosclerosis. Finally, we provide three novel and simple interaction models between lncRNAs and RBPs.

## The Biology of Long Non-coding RNA and RNA-Binding Protein

lncRNAs are defined as non-coding transcripts of more than 200 nucleotides in length, that do not translate into functional proteins (4), and they have low conservation with species (13). Based on the origin from different genomic locations, we classify lncRNAs are classified into intergenic lncRNAs (also known as lincRNA), intronic lncRNAs, sense lncRNAs, and antisense lncRNAs (14). Elements that determine the extent of lncRNA expression include core promoters, enhancers, and transposable elements (15). Like coding protein genes, most lncRNAs are transcribed by RNA polymerase II, but the promoters of non-coding protein genes have fewer overlapping transcription factor binding motifs and therefore give low levels of lncRNA expression (15). Moreover, many lncRNAs can be produced from enhancers, which are genomic binding sites encoding sequence-specific activator or repressor transcription factors (TFs) regions, and these elements often confer more tissue-specific expression (15). Transposable elements (TEs) are also an important component of lncRNAs biology. Approximately 75% of lncRNAs transcripts contain sequence elements derived from TEs. In addition, 25% of TEs overlap with the transcription start site (TSS) and poly (A) signal (PAS) of lncRNA genes (16). Thus, they are important drivers of lncRNA expression. The localization of lncRNAs within the cell determines its function (17). lncRNAs have been found to exist in the nucleolus, chromatin speckles, and paratopes (18). In addition, some lncRNAs can be transferred into the cytoplasm, further be selectively localized in the mitochondria, ribosomes, extracellular membranes, and exosomes (11).

Accumulated evidence shows that lncRNAs can bind to DNA, RNA, and proteins; change the stability and translation of cytoplasmic mRNAs; and interfere with signaling pathways (4). According to the molecular mechanism of action, lncRNAs can be divided into three subgroups: (1) lncRNA loci containing enhancers that regulate gene expression; (2) lncRNA loci whose transcriptional behavior, rather than the transcript itself, has an important role in regulating neighboring genes; and (3) lncRNA transcripts that achieve their cellular functions by interacting with DNA, other RNAs, and proteins (19, 20). However, the mechanism of lncRNA directly interacting with RNA-binding proteins have attracted our attention.

RNA-binding proteins are proteins that bind RNA through one or more RNA-binding domains (RBDs) and alter the fate or function of the bound RNA, its activity, or the expression of the target gene (21). The structures and mechanisms by which RBP binds and regulates RNA are very diverse (22). Because the structure and function of RBDs provide some insights about the binding preference and target of RBP, it is usually classified according to its specific RBDs (10). Normally, RBPs assemble with RNA to form ribonucleoprotein particles (RNPs) that mature, process, regulate, or transport RNAs (10, 23). The RBDs are the functional unit of protein-bound RNA (22). Most RBPs contain an RNA recognition motif (RRM), a K homology (KH) domain, a DEAD motif, a double-stranded RNA-binding motif (DSRM), or a zinc-finger domain (10). Additionally, some RBPs lacking conventional RNA-binding domains have been discovered (21, 22). As an RBP that needs to typically bind to AU-rich elements (ARE, core sequence 5′-AUUUA), thereby govern the fate of mRNA transcripts from biogenesis, stabilization, translation to RNA decay (24). Based on target-RNA categorization, among the 20,500 protein-coding genes in humans, nearly 39% of RBPs were involved in the non-coding RNA metabolic processes (10).

Similar to lncRNAs, RBP regulatory function in atherosclerosis has also garnered attention. Therefore, understanding the regulatory molecules of lncRNAs and RBPs in atherosclerosis as well as the interaction mode between lncRNA and RBP will lay a foundation for future understanding and prevention and treatment of atherosclerosis from the perspective of molecular biology.

## lncRNA and RBP as Regulators of Atherosclerosis

There is increasing evidence that lncRNAs and RBPs can serve as important regulators of cellular function in atherosclerosis. It is reported that lncRNA ANRIL was the first non-coding RNA identified to be associated with atherosclerosis and expressed in endothelial cells, smooth muscle cells, and immune cells (25). Nevertheless, the regulatory mechanisms of non-coding RNA in atherosclerosis were poorly studied at that time. Subsequent researchers verified that ANRIL can promote cell proliferation, migration, and inhibit apoptosis through various mechanisms, such as trans-regulation of target genes or spongy miR-399-5p, and regulation of the RAS/RAF/ERK signaling pathway (26–30). The research on regulatory mechanisms of lncRNAs in atherosclerosis has started a boom. Additionally, through a PubMed search from March 2012 through March 2021, many lncRNAs related to atherosclerosis have been discovered in the past 10 years. We have summarized their regulatory mechanisms in detail in Table 1. However, RBPs have yet to be extensively researched in atherosclerosis. The few studies that have investigated RBPs involved in atherosclerosis suggest an important role in proliferation, migration, and apoptosis. and, a small amount of RBPs have been shown to regulate cholesterol homeostasis. For example, the RBP VIGILIN can regulate the hepatic very low-density lipoprotein (VLDL) secretion, and inhibition of VIGILIN decreases hepatic VLDL secretion and circulating LDL-C levels (92). By retrieving information from PubMed up to January 2021, we summarize RBPs' regulatory mechanisms as follows (Table 2). Given that ECs, monocytes/macrophages, and VSMCs are crucial in the development of atherosclerosis, we have summed up the significance of lncRNAs and RBPs in each of these cells below.

**Table 1:** Long non-coding RNAs in atherosclerosis-related research.

**lncRNAs**	**Endothelial cells**	**Smooth muscle cells**	**Monocytes/macrophages**	**Atherogenic/atheroprotective**	**Experimentally validated function**	**References**
ANRIL	Yes	Yes	Yes	Atherogenic	Promote cell proliferation, adhesion, migration, and decrease apoptosis	(28–30)
DYNLRB2-2	No	No	Yes	Atheroprotective	Promote cholesterol efflux and inhibit inflammatory response	(31)
lincRNA-p21	No	Yes	Yes	Atheroprotective	Inhibit cell proliferation and induce apoptosis	(32)
RP5833A20.1	No	No	Yes	Atherogenic	Affect cholesterol homeostasis and inflammation	(33)
HOXC-AS1	No	No	Yes	Atheroprotective	Suppress Ox-LDL-induced cholesterol accumulation	(34)
HULC	Yes	No	No	Atherogenic	Regulate TNF-α-induced apoptosis	(35)
RNCR3	Yes	Yes	No	Atheroprotective	Decrease proliferation, migration, and accelerate apoptosis	(6)
HOTAIR	Yes	No	Yes	Atheroprotective	Regulate the EC proliferation and migration/aggravate oxidative stress and inflammation response in macrophages	(36, 37)
LINC00305	No	Yes	Yes	Atherogenic	Promote monocyte inflammation and induce HASMC phenotype switching	(38)
TUG1	No	Yes	No	Atherogenic	Promote proliferation, migration, invasion, and metastasis	(39, 40)
MALAT1	Yes	No	No	Atheroprotective/ atherogenic	Suppress inflammatory cytokine release, apoptosis, and promote EndMT	(41, 42)
DYN-LRB2-2	No	No	Yes	Atheroprotective	Upregulate cholesterol efflux	(43)
DIGIT	Yes	No	No	Atheroprotective	Promote growth, migration, and tube formation	(44)
MeXis	No	No	Yes	Atheroprotective	Promote cholesterol efflux	(45)
UCA1	No	Yes	No	Atherogenic	Regulate migration and proliferation	(46)
XIST	Yes	No	No	Atherogenic	Regulate the expression of NOD2	(47)
SRA	Yes	No	No	Atherogenic	Repress inflammatory-related cytokines	(48)
ENST00113	Yes	Yes	No	Atherogenic	Promote proliferation, survival, and migration	(49)
GAS5	Yes	No	Yes	Atherogenic	Aggravate inflammatory response, MMP expression, autophagy dysfunction, apoptosis	(50–52)
H19	No	Yes	No	Atherogenic	Promote proliferation and anti-apoptosis	(53)
MEG3	Yes	Yes	No	Atherogenic	Enhance pyroptosis and modulate proliferation and apoptosis balance	(54, 55)
MIAT	Yes	Yes	No	Atherogenic	Promote proliferation, angiogenesis, inflammatory factors expression, and hinders apoptosis	(56, 57)
SNHG16	No	Yes	Yes	Atherogenic	Promote proliferation, migration, and inflammatory response	(58, 59)
430945	No	Yes	No	Atherogenic	Promote proliferation and migration	(60)
FA2H-2	Yes	Yes	No	Atheroprotective	Suppress MLKL expression, activate autophagy, and restrain inflammation	(61)
NEXN- as1	Yes	Yes	No	Atheroprotective	Inhibit TLR4 oligomerization and NF-κB activity	(62)
CCL2	Yes	No	No	Atherogenic	Positively regulate CCL2 mRNA levels	(63)
CDKN2B-AS1	No	No	Yes	Atheroprotective	Reduce inflammatory response and promote cholesterol efflux	(64)
Linc00299	Yes	Yes	No	Atherogenic	Increase proliferation, migration, and inhibit apoptosis	(65)
AF131217.1	Yes	No	No	Atheroprotective	Inhibit inflammation	(66)
APTR	Yes	No	No	Atherogenic	Elevate proliferation, migration, and pipe-formation	(67)
RAPIA	No	No	Yes	Atherogenic	Promote proliferation and reduce apoptosis	(68)
SNHG12	No	Yes	No	Atherogenic	Promote proliferation and migration	(69)
CASC11	No	Yes	No	Atheroprotective	Downregulation of IL-9, proliferation, and promote apoptosis	(70)
NEAT1	No	No	Yes	Atherogenic	Increase inflammation response and lipid uptake	(71)
MEG8	No	Yes	No	Atheroprotective	Suppress proliferation, migration, and induce apoptosis	(72)
LEF1-AS1	No	Yes	No	Atherogenic	Regulate migration and proliferation	(73)
DAPK-IT1	No	No	Yes	Atherogenic	Regulate cholesterol metabolism and inflammatory response	(74)
HOXA-AS3	Yes	No	No	Atherogenic	Promote NF-κB-mediated endothelium inflammation	(75)
NORAD	Yes	No	No	Atheroprotective	Attenuate senescence, apoptosis	(76)
LOC285194	No	Yes	No	Atherogenic	Promote proliferation, invasion, migration, and inhibit apoptosis	(77)
HOTTIP	No	Yes	No	Atherogenic	Promote proliferation and migration	(78)
Linc-ROR	No	Yes	No	Atherogenic	Promote proliferation and migration	(79)
HCG11	No	Yes	No	Atherogenic	Promote the proliferation and inhibit apoptosis	(80)
MAARS	No	No	Yes	Atherogenic	Induce apoptosis	(81)
XXYLT1-AS2	Yes	No	No	Atheroprotective	Inhibit proliferation and migration	(82)
CTBP1-AS2	No	Yes	No	Atheroprotective	Inhibit proliferation and autophagy	(83)
FOXC2-AS1	No	Yes	No	Atherogenic	Promote proliferation and inhibit apoptosis	(84)
LINC00657	Yes	No	No	Atherogenic	Induce endothelial cell injury	(85)
SENCR	No	Yes	No	Atheroprotective	Inhibit proliferation, migration, and block cell cycle	(86)
kcnq1ot1	No	No	Yes	Atherogenic	Promote lipid accumulation	(87)
PEBP1P2	No	Yes	No	Atheroprotective	Decrease proliferation and migration	(88)
SNHG7	Yes	No	No	Atheroprotective	Repress proliferation and migration	(89)
RP11-728F11.4	No	No	Yes	Atherogenic	Increase intracellular cholesterol accumulation and proinflammatory cytokine	(90)
SMILR	No	Yes	No	Atherogenic	Promote proliferation	(91)

**Table 2:** RNA-binding proteins in atherosclerosis-related research.

**RBPs**	**RNA interactors**	**Endothelial cells**	**Smooth muscle cells**	**Monocyte/macrophages**	**Atherogenic/atheroprotective**	**Experimentally validated function**	**References**
HUR	LncRNA MAARS/ABCA1 mRNA	No	No	Yes	Atherogenic	Induce apoptosis and promote cellular cholesterol efflux	(81, 93)
STAU1	LncRNA SMILR	No	Yes	No	Atherogenic	Degrades the SMILR: CENPF interaction to mediate VSMC proliferation	(91)
QKI-7	mRNACD144/ NLGN1/ TSG6	Yes	No	No	Atherogenic	Defective EC barrier function and compromised angiogenesis	(94)
MEX3A	MIR126-5p	Yes	No	No	Atheroprotective	Inhibit proteolytic activity and limit apoptosis	(95)
IGF2BP1	EV-derived miR-146a	No	No	Yes	Atherogenic	Decrease migration and promote macrophage entrapment	(96)
EWSR1	lncRNA RP11-728F11.4	No	No	Yes	Atherogenic	Increase intracellular cholesterol accumulation and proinflammatory cytokine	(90)
LARP6	COL1a1/COL1a2 mRNA	No	Yes	No	Atherogenic	IGF-1 enhances collagen fibrillogenesis *via* induction of LARP6	(97)
FUS	lncRNAXXYLT1-AS2	Yes	No	No	Atheroprotective	Inhibit proliferation and migration	(82)
KSRP	miR-185	No	No	No	Atherogenic	Negatively regulate the expression of LDLR in hepatic cells to control cholesterol homeostasis	(98)
VIGILIN	Apolipoprotein B /Apob mRNA	No	No	No	Atherogenic	Increase VLDL/LDL levels and formation of atherosclerotic plaques	(92)
ZFP36	IL-6/MCP-1 mRNA	Yes	No	Yes	Atheroprotective	Regulate MCP-1 and IL-6 mRNA stability and reduce its expression	(99)
hnRNPL	linc-AAM	No	No	Yes	Atherogenic	Activate macrophages and promote the immune response	(100)
TTP	NLRP3 mRNA	No	No	Yes	Atheroprotective	As a negative regulator of NLRP3 inflammasome	(101)

### Endothelial Cells

The vascular endothelium is a single layer of ECs that constitute the intima of arteries, veins, and capillaries (102). It is widely acknowledged that EC dysfunction is a key step in atherosclerosis initiation (103). Endothelial stimulation by NF-κB signaling increases the expression of EC adhesion molecules and promotes monocyte recruitment in the vessel wall, which can cause oxidative stress and promote the progression of inflammation (103). A large and growing body of literature has reported that lncRNA plays a role in ECs. Analysis of human umbilical vein endothelial cells after ox-LDL stimulation by lncRNA expression microarray reveals a large number of differentially expressed lncRNAs (104). Clopidogrel, a commonly antiplatelet medication, has been found to inhibit the expression of lncRNA HIF1A-AS1 to reduce EC injury (105).

More recently, several published studies have described that lncRNAs may contribute to their role in inflammation, proliferation, migration, and apoptosis. For example, Hu et al. observed that the expression of NEXN was upregulated by lncRNA NEXN-AS1 (62). Prior studies found that initiation of the TLR4/NF-κB signaling pathway would induce the expression of inflammatory molecules such as MCP1, TNF-α, and IL-6 (62, 106, 107). Theoretically, however, NEXN is a filamentous actin-binding protein (108), which can cause TLR4/NF-κB inactivity, diminish inflammatory molecules, suppresses monocyte recruitment, and prevent atherosclerosis (62). Experimental studies have also found that lncRNAs can regulate EC gene expression; for instance, lncRNA-CCL2 upregulates the levels of its adjacent CCL2 gene, which is a pro-atherosclerotic chemokine gene in the EC lines. Further, the lncRNA APTR can promote the proliferation and migration of ECs (67). HULC can promote the apoptosis of ECs (35). These are the interesting examples used to demonstrate that lncRNA regulates atherosclerotic ECs. Overall, many more studies have confirmed the value of lncRNAs to ECs, even though other mechanisms are not yet clear and need further study in the future.

Similar to lncRNAs, evidence has shown that RBPs play important roles in EC function. For example, the RBP QKI-7 promotes degradation of CD144/NLGN1/TSG6 mRNA, which plays a negative role in EC barrier function and angiogenesis, thus aggravating the progress of atherosclerosis (94). More recently, a variety of RBPs have been shown to affect autophagy, apoptosis, proliferation, and migration of ECs. MEX3A is an RBP that can form a ternary complex with AGO2 on the autophagosome surface and facilitates its nuclear localization to inhibiting proteolytic activity and limiting apoptosis (95). Another interesting study revealed that FUS can also regulate endothelial function. FUS is an atheroprotective factor that inhibits EC proliferation and migration by directly interacting with the lncRNAXXYLT1-AS2. If XXYLT1-AS2/FUS is inhibited, the expression of adhesion molecules (VCAM-1) and chemoattractant proteins (MCP-1) is increased, and monocytes can more easily adhere to ECs (82). The growing evidence reported here draws attention to the contributions of RBPs, particularly in human EC models. However, the significance of these RBPs to whole organism development and function is less well-understood.

### Macrophages

During the development of atherosclerosis, macrophages differentiate from monocytes entering the intima and become macrophage-derived foam cells by taking up ox-LDL (109). At the same time, macrophages further secrete cytokines such as inflammatory cytokines (IL-1, IL6, TNF) and chemokines (CCL2, CCL5, CXCL1), and protein hydrolases to exacerbate plaque inflammation by recruiting reinforcements such as monocytes, neutrophils, and T-lymphocytes (110, 111). Macrophage are also defined as an immune cell that can express pattern recognition receptors (PRR) including NOD-like receptors, scavenger receptors, and Toll-like receptors, and are often triggered by “damage” signals from ox-LDL (109, 112). The proliferation of inflammatory macrophages and phenotypic switching are the main factors involved in the progression of atherosclerosis. Macrophages can shift toward a pro-inflammatory phenotype that we call M1 type, or to an anti-inflammatory phenotype that we call M2 type (112).

When macrophages are activated, they trigger an innate immune response. IFN-γ which belongs to Th1 cytokines and lipopolysaccharides can activate M1 macrophages to make pro-inflammatory cytokines. These proinflammatory cytokines contain TNF-α, IL-1β, IL-6, IL-12, and IL-23 (113). M2 macrophages are stimulated by Th2 cytokines that include IL-4 and IL-13, and further produce anti-inflammatory cytokines such as TGF-β and IL-10 (113, 114). Macrophages differentiate into different morphological and functional phenotypes according to changes in the microenvironment (115, 116).

Macrophage polarization is a plastic process. Pathways that have been described in the regulation of macrophage polarization include the PI3K/Akt pathway, Notch pathway, JAK-STAT pathway, TGF-β signal pathway, and Wnt/β-catenin pathway, among others. The PI3K/Akt pathway is activated by TLR4 and other pathogen recognition receptors, cytokines and chemokines, and Fc receptors, which regulate downstream signals that control cytokine production. Akt promotes M2-type macrophage polarization and inhibits M1-type polarization. It is essential for the anti-inflammatory response (117). Signal regulatory protein (SIRP) α is abundantly expressed in macrophages, and plays a critical role in regulating innate immune activation, and can as a novel target of Notch-mediated macrophage polarization (118). SOCS proteins function as feedback inhibitors for cytokines that use the JAK/STAT pathway, given that SOCS3 deficiency, IFN-γ-induced STAT1 and STAT3, and GM-CSF–induced STAT5 will be activated, promote M1 polarization (119). Conversely, IL-4 not merely activates STAT6 but promotes the differentiation of TH2 cells that stimulate M2 macrophage responses (120). Therefore, through the JAK/STAT pathway, cytokines can lead to the activation of transcription factors that dictate M1/M2 polarization and mediate inflammatory responses in macrophages. Likewise, Interleukin-4 (IL-4) regulates macrophages polarization *via* the TGF-β1/Smad pathway (121). The Wnt/β-catenin pathway activation promotes differentiation of macrophages in the M2 direction, which exhibits anti-inflammatory activity (122).

Increasing evidence suggests that lncRNAs play crucial roles during the differentiation of monocyte/macrophage, proliferation, decay, and phenotypic switching of macrophages. However, our insight into the contribution of lncRNAs is still in the early stages. For example, gene overexpression and knockdown experiments show that lncRNA MALAT1 has a positive effect on the pyroptosis of normal macrophages, and downregulation of lncRNA-MALAT1 can abate the pyroptosis of macrophages in rats with diabetic atherosclerosis (123). Additionally, LXR, a ligand-activated nuclear receptor, is also a transcription factor that regulates the expression of genes related to the macrophage's response to cholesterol, including ABCA1, which encodes the plasma membrane transport protein ABCA1, to promote macrophage cholesterol efflux. Sallam et al. found that the LXR-activated lncRNA Mexis increased ABCA1 protein expression levels while enhancing cholesterol efflux (45), which provides novel insights into the prevention and treatment of atherosclerosis. Similarly, the lncRNA DYNLRB2-2 exerts its atheroprotective effect by increasing cholesterol efflux and decreasing inflammatory responses (31). lncRNA HOXC-AS1 can prevent atherosclerosis by decreasing cholesterol accumulation by the mechanism of upregulating HOXC6 expression (34).

Additional studies have revealed that RBPs play a vital role in macrophage migration, apoptosis, and cholesterol regulation and is a crucial driver of atherosclerosis and integration factors of metabolic and inflammatory signals. RBP IGF2BP1 interacts with extracellular vesicle-derived miR-146a to decrease cell migration and promotes macrophage entrapment (96). A recent study identified that HuR not only interacts with ABCA1mRNA to promote cellular cholesterol efflux (93); but also interacts with lncRNA MAARS in the macrophage nucleus, preventing its shuttling to the cytosol and interfering with its RNA-stabilizing function to induce apoptosis (81). The RNA-binding protein tristetraprolin (TTP, encoded by ZFP36) has been found early in eukaryotes (124). This was characterized by the RNA-binding tandem zinc finger (TZF) domain and often be associated with cancer, as well as other inflammatory diseases (101, 125–127). Its function is as a decay signal of RNA by binding to AREs, and adenylate/uridylate-rich RNA motifs (128). As is known, the NLRP3 inflammasome drives the progression of atherosclerosis. Studies have shown that TTP possibly binds to a main ARE and inhibits the expression of NLRP3 in macrophages and other cell types, while, directly affecting TTP expression (101). However, its main test in animal experiments, further research some wonderful strategies to targeted therapy are promising.

### Smooth Muscle Cells

It is known that VSMCs are the pivotal cells in the media layer of arteries. Their effects include regulation of arterial contraction, compliance, and production of extracellular matrix (ECM) (129, 130). VSMCs are essential in the stages of atherosclerotic plaque formation and are beneficial and essential for plaque stability (129, 131). Ox-LDL, proinflammatory cytokines, high levels of nitric oxide (NO), and mechanical damage can induce VSMC apoptosis. Furthermore, VSMC apoptosis contributes to plaque inflammation (131). As mentioned before, ox-LDL can stimulate VSMC necrosis and highly oxidized LDL induces necrosis, while mildly oxidized LDL induces ER stress/apoptosis (131). ECs, macrophages, and VSMCs may present autophagic activation in human atherosclerotic plaques, but this needs validation in further studies (131, 132). During atherosclerosis, VSMCs undergo complex structural and functional changes that produce a wide range of phenotypes, including foam cell formation (133).

Published studies have identified many lncRNAs that play a role in controlling VSMCs proliferation, migration, and apoptosis. For example, lncRNA SNHG16 regulates smooth muscle cell proliferation and migration through sponging miR-205 and regulating Smad2. Overexpression of SNHG16 promotes VSMC proliferation and migration, whereas downregulation of SNHG16 inhibits PDGF-bb-stimulated VSMC proliferation and migration (59). Furthermore, the imbalance between proliferation and apoptosis of VSMCs also plays a crucial role during the early stage of atherosclerosis. Li et al. identified lncRNA-MEG3 as a crucial regulator in the balance between proliferation and apoptosis of VSMCs, which could sponge miR-26a as a competing endogenous RNA (55). In addition to lncRNA-MEG3, Chen et al. revealed that targeting LOC285194 can boost cell proliferation and obstruct apoptosis (77). Another novel regulatory factor, lncRNA 430945, significantly suppressed VSMC proliferation and migration *via* ROR2 (60).

There are fewer RBPs that were found to play a regulatory role in atherosclerotic SMCs than lncRNAs. RNA-sequencing identified smooth muscle–induced lncRNA (SMILR) as a novel intergenic lncRNA activated by VSMCs proliferation. Recent evidence suggests that SMILR interacts with CENPF mRNA and STAU1 in the Cell Cycle Network (91). Because STAU1 is a universally expressed and multifunctional RNA-binding protein, STAU1 participates in mRNA transport and localization to mediate further translation (134).

Taken together, these studies show that lncRNAs are crucially important in the proliferation, migration, apoptosis, and senescence of ECs, VSMCs, and monocytes/macrophages, resulting in two different outcomes of promoting the progression of atherosclerosis or protecting from atherosclerosis. Given the recent curiosity in relating proliferative pathways to plaque development, lncRNAs, and RBPs may be a hopeful direction.

## Emerging RBPs Interact With lncRNA in Atherosclerosis

**Figure 1:**
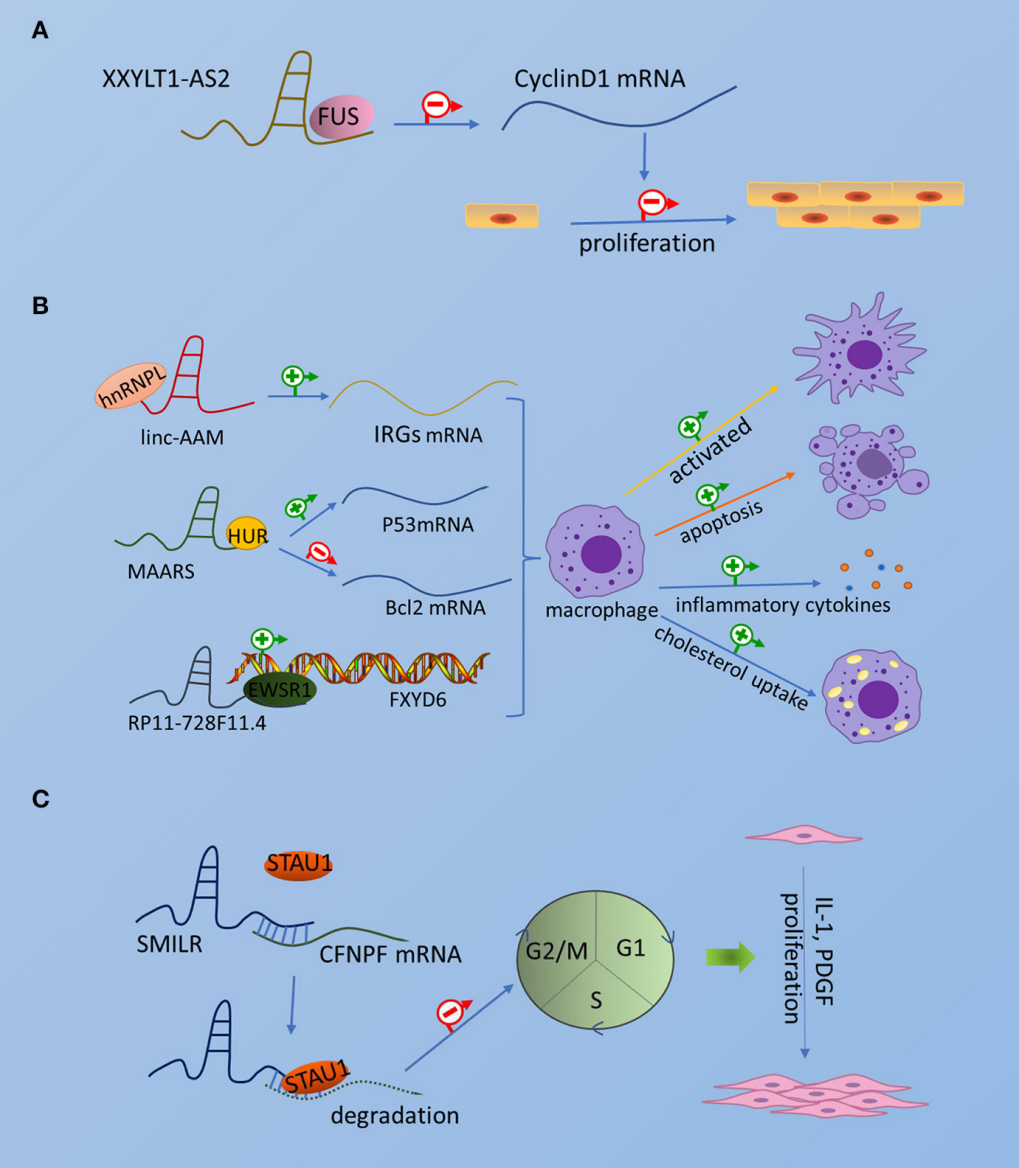
The interaction of lncRNA and RBP in atherosclerosis. The picture schematically represents the interaction of emerging RBPs and lncRNAs in atherosclerotic endothelial cells, smooth muscle cells, and macrophages. **(A)** Endothelial cells. **(B)** Smooth muscle cells. **(C)** Macrophages.

Thus far, few RBPs associated with atherosclerosis have been found to interact with lncRNAs to exert regulatory functions. Although these RBPs are not particularly well-reported, it is worthwhile to further investigate the related aspects of the mechanism, which will be beneficial to provide a basis for future disease treatment through etiology. Collectively, the interaction of lncRNA and RBP in atherosclerosis is shown in Figure 1.

### Human Antigen R

HuR (also known as Elavl1) is an RBP that is essential in cellular responses to the immune system and cell cycle (135) and has anti-apoptotic functions (136). Studies have found that HuR is expressed in endothelial cells, VSMCs, and macrophages in the atherosclerotic plaque (137). Nevertheless, the role of HuR in atherosclerosis may be highly dependent on the cell type specificity. According to Feinberg et al. (81), lncRNA MAARS can interact with HuR/ELAVL1, 14 HuR-specific AREs have been identified in the MAARS transcript and the ARE-specific binding of MAARS to HuR was confirmed. The interaction of lncRNA MAARS and HuR can induce macrophage apoptosis and decrease their efferocytosis in advanced plaques by regulating HuR targets such as p53, p27, caspase-9, and Bcl2 to alter HuR cytosolic shuttling. Interestingly, Zhang et al. (138) used smooth muscle-specific HuR knockout mice (HuRSMKO) to investigate the function of HuR in atherosclerosis. The plaque load was increased in the HuRSMKO mouse model of atherosclerosis compared with controls. It was verified that HuR could bind mRNA of adenosine 5-monophosphate-activated protein kinase (AMPK) α1 and AMPKα2, thus improving their stability and translational ability. In contrast, HuR deficiency lead to decreased p-AMPK and LC3II levels and increased p62 levels, resulting in defective autophagy. In turn, AMPK activation induces autophagy and inhibits atherosclerosis in HuRSMKO mice. However, previous studies reported a novel mechanism by which the anti-inflammatory cytokine IL-19 can decrease HuR mRNA expression, leading to decreases in mRNA stability of pro-inflammatory cytokines, to mediated atheroprotective effects. However, the lack of IL-19 leads to increased atherosclerosis (137). In addition, studies have revealed the role of endothelial HuR deficiency in attenuating atherosclerosis, and this effect may be partly due to the decreased expression of proatherogenic molecules and suppressed local inflammation (139). Collectively, HuR as direct or indirect regulators in atherosclerosis is a complex concept and needs to be further explored.

### Heterogeneous Nuclear Ribonucleoproteins

The hnRNPs are a type of ribonucleoprotein (RNP) and belong to a large family of RBPs that play an essential role in the cellular nucleic acid metabolism. Their function varies depending on the cellular localization (140, 141). The hnRNPs family has many members such as hnRNPA/B, hnRNPC, hnRNPD, hnRNPE, hnRNPF/H, hnRNPG, hnRNP I (PTBP1)/L, hnRNPK, and hnRNPM/Q, hnRNP P2 (FUS/TLS), and hnRNP R/U and they have different and complex functions (140). The reported functions are as follows: alternative splicing, formation of RNP complex with pre-mRNA, mRNA stabilization, RNA transport, transcriptional and translational regulation, and inhibition of cell differentiation (140, 142). Nuclear lncRNAs interact with heterogeneous nuclear ribonucleoproteins in regulating cellular functions such as glucose and lipid metabolism, immune response, DNA damage response, and others (141).

In an atherosclerosis model, the level of hnRNPK protein was elevated in SMCs, and its subcellular localization was related to the cell cycle. Early in the cell cycle, a slight increase in cytoplasmic hnRNPK may be associated with increased neogenesis, but at the end of the cell cycle, hnRNPK accumulated in the cytoplasm and decreased in the nucleus, indicating translocation of nuclear hnRNPK to the cytoplasm and suggesting that hnRNPK regulates vascular smooth muscle proliferation (143). Chen et al. found that linC-AAM can interact with the RBP hnRNPL through the CACACA motif to activate macrophages and promote immune response; In their experiments, they localized the lncRNA in the nucleus by RNA fluorescence *in situ* hybridization (FISH) and detected hnRNPL by RNA pull-down assay and confirmed its activation of macrophages and promotion of immune response gene expression by linc-AAM silencing or knockout (KO) and overexpression of lncRNA (100). Studies of hnRNPC expression in atherosclerosis are particularly scarce. A previous study found that hnRNP is mainly expressed in ECs and that hnPRNC is involved in vascular cell signaling pathways activated by low physiological levels of hydrogen peroxide, which can regulate vascular cell proliferation (144). The hnRNP P2 as the RBP fused in sarcoma/translocated in liposarcoma (FUS/TLS), is also called FUS, which mutants often be reported to relate to cancer and neurodegeneration in humans (140, 145). Recently, a study found that lncRNA that interacts with FUS can regulate proliferation and migration of ECs in atherosclerosis (82).

### Ewing Sarcoma Breakpoint Region 1/EWS RNA Binding Protein 1

EWSR1 plays an important role in neurodegeneration, epigenetic alteration, and cellular functions such as autophagy and mitochondrial activity (146). A growing body of evidence indicates that lncRNA is an important player in atherosclerosis. For example, researchers revealed that lncRNA RP11-728F11.4 interaction with the RNA-binding protein upregulated the cognate gene FXYD6 in atherosclerotic plaques. Knockdown or overexpression of RP11-728F11.4 affected cholesterol uptake, inflammatory molecule production, levels of lipids in monocytes-derived macrophage (90). Hence, EWSR1 is also a key regulator in atherosclerosis.

### Staufen 1

STAU1 is a double-stranded (ds) RNA-binding protein known to be involved in mRNA decay. It binds dsRNA structures that are formed not only by intramolecular base-pairing of 3′UTR sequences but also by intermolecular base-pairing of 3'UTR sequences with a lncRNA *via* partially complementary Alu elements (134). The lncRNA SMILR is a novel intergenic lncRNA activated by VSMC proliferation and is related to atherosclerosis. It was shown to interact with centromere protein F (CENPF) mRNA to promote VSMC proliferation. Interestingly, using RNA pull-down and mass spectrometric analysis, STAU1 was found likely to bind to SMILR within the first half of its sequence, which is the predicted interaction site with CENPF. Knockdown of STAU1 upregulates the expression of SMILR and CENPF mRNA (91).

The structures and mechanisms *via* which RNA-binding proteins interact with transcripts are varied and complex. Not to mention the binding to lncRNAs, because lncRNAs are involved in a variety of biological functions.

## The Interaction ModeL of lncRNA and RBP

### RBPs Regulate the Expression of lncRNAs

Degradation, repression and overexpression of lncRNAs are important for the regulation of biological messaging; however, in addition to physiological factors (infection, tumor) (147, 148), physical factors (temperature) (149), that has been found to affect lncRNA normal expression. A few known lncRNAs have been reported to be affected by their binding protein. For example, Bachand et al. identified a class of poly(A)-binding protein nuclear 1(PABPN1) sensitive lncRNAs, PABPN1 promotes post-transcriptional regulation of sensitive lncRNAs through polyadenylation (150). Similarly, the serine/arginine-rich splicing factor 1 (SRSF1) plays a positive role on the regulation of lncRNA NEAT1 in gliomas (151). Based on RNA-Binding Protein Immunoprecipitation (RIP) analysis, the RBP SRSF1 directly interacts with NEAT1, if knock down the SRSF1, the NEAT1 would fast degradation (151). There are also reports that HuR plays a similar role in lncRNA NEAT1 (152). In short, RBPs can regulate the stability of lncRNA. Furthermore, the study found that PTB-associated splicing factor (PSF) a protein that has both RNA-binding domains and DNA-binding domains, binds to and represses the lncRNA CTBP1-AS promoter. CTBP1 expression is generally upregulated in prostate cancer, and they could be promising targets for therapeutic options of prostate cancer (153). Furthermore, Tian et al. found that TTP regulates lncRNA HOTAIR expression by a posttranscriptional mechanism. HOTAIR is a downstream target of TTP, and according to the AUUUA consensus sequence, the researcher predicted four TTP-binding sites of HOTAIR. They also used RIP to investigate the role of TTP in the regulation of HOTAIR expression (154).

Taken together, the expression of RBPs effect the level of lncRNAs, but the mechanism is yet unclear and needs further investigation, the same RBPs can regulate different lncRNAs, by contrast, the same lncRNA can be regulated by different RBPs in a different environment.

### lncRNAs Regulate the Target Gene Expression by Interacting With RBPs

In addition to binding to lncRNAs to affect the expression of lncRNAs, RBPs can also affect the expression of lncRNA target genes. For example, Chen et al. found that exosomal lncRNA LNMAT2 recruited hnRNPA2B1 to the PROX1 promoter to upregulate PROX1 expression by directly interacting with hnRNPA2B1, leading to lymphangiogenesis and lymphatic metastasis in bladder cancer (155). The exosome-mediated lncRNA AFAP1-AS1 can bind to AUF1 and activate ERBB2 translation to regulate the resistance of trastuzumab (156). The lncRNA RP11-728F11.4 was shown to interact with the RBP EWSR1 and upregulate the expression of the homologous gene FXYD6, which encodes a Na+/K+- ATPase regulator and Na+-ATPase. The increased activity of Na+-ATPase, intracellular cholesterol accumulation, and pro-inflammatory cytokine production increased atherosclerotic lesions (90).

The molecular mechanisms by which lncRNAs bind to RBP to affect the expression of lncRNA target genes are complex and much is still unknown. We know that lncRNA can act as a scaffold, specifically, lncRNA acts as a structural component for nucleic acid-protein (also name ribonucleoprotein) complexes formed with proteins to connect multiple proteins to regulate the expression of their target genes. For instance, hnRNPK and YBX1 are lncRNA SCAT7-interacting proteins, and recruitment of the FGFR2 and FGFR3 promoter regions SCAT7-hnRNPK-YBX1 RNP complexes promotes transcriptional activation of the FGF/FGFR pathway, resulting in sustained cell proliferation *via* the PI3K/AKT and Ras/MAPK pathways (157). Further, hnRNPK has been reported to bind to lincRNA-p21 to repress the target p53 transcription (158).

### lncRNAs Regulate the Activity of Their Specific Binding Proteins

**Figure 2:**
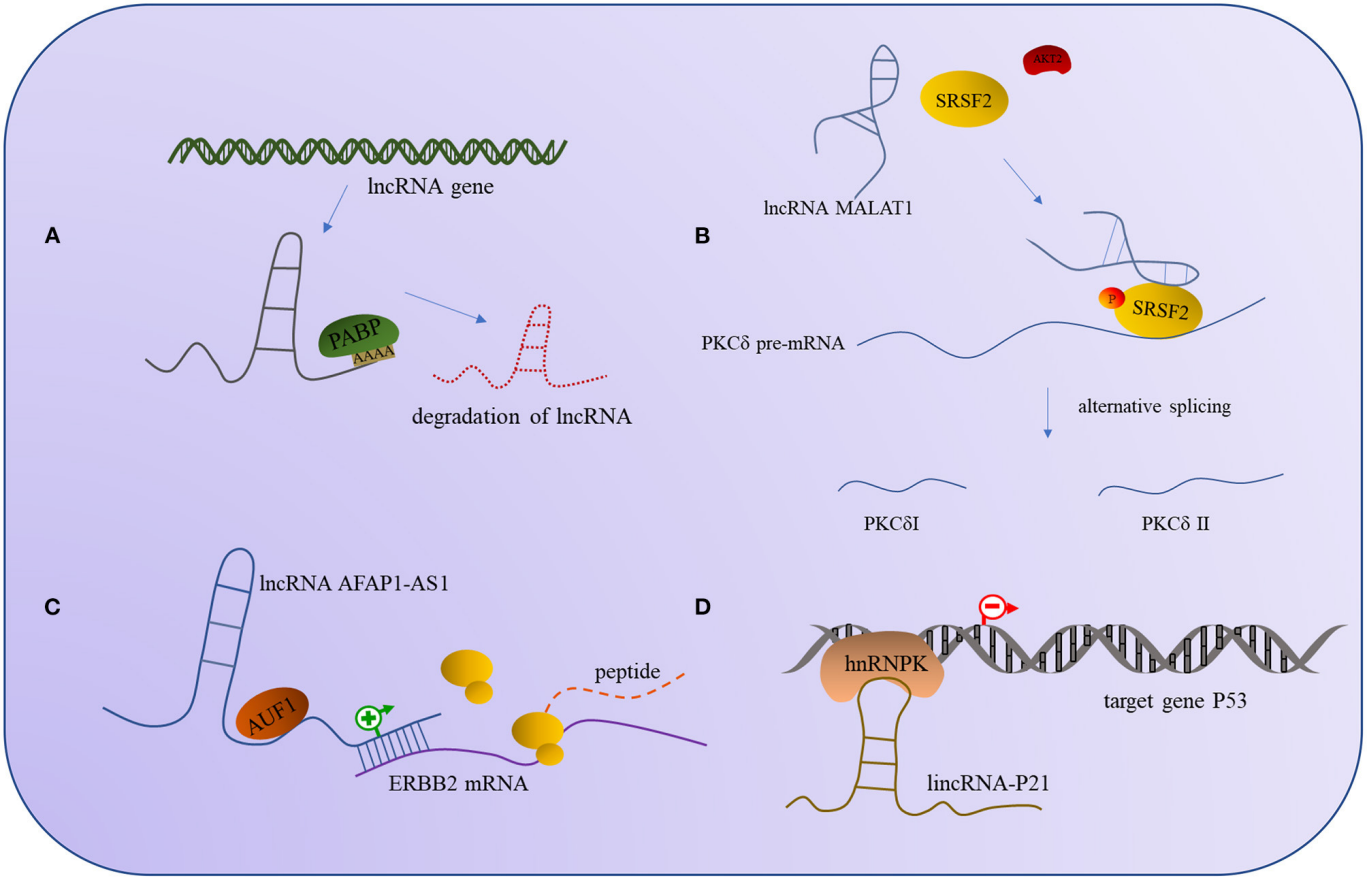
The interaction models between lncRNA and RNA-binding protein. **(A)** PABP interacts with lncRNA induces the degradation of lncRNA. **(B)** The interaction of lncRNA AFAP1-AS1 and RBP AUF1 can promote the expression of ERBB2 mRNA. **(C)** The combination of lncRNA MALAT1 and SRSF2 can promote the phosphorylation of SRSF2 by AKT2 to alternative splicing PKCδ. **(D)** The interaction of hnRNPK and lincRNA-P21 can repress the expression of the target gene P53.

The binding of lncRNA to RBP can regulate the expression of lncRNA and affect the expression of the target gene, and it can also affect the activity of the binding proteins; the outcomes are similar, in that all of them will affect the corresponding signaling pathway and thus change the regulatory outcome of the molecule. For example, the interaction between lncRNA and UPFI can regulate the expression of other mRNA but also decrease the stability of the lncRNAs. In some cases, the binding of lncRNA and UPF1 will affect the expression of UPF1, though the specific mechanisms involved need to be further investigated (159). Additionally, the lncRNA MALAT1 recruits SRSF2 and binds tightly to it, thus making the AKT2 (serine/threonine kinase2) effectively phosphorylate SRSF2 and form a stable combination of PKCδ pre-mRNA, promoting selective splicing of PKCdII in HT22 cells (160). Apart from phosphorylation modification to alter protein activity, ubiquitination is also a common modality. Studies have found that lncRNA mamRNA supports antagonistic RBPs Mmi1 and Mei2 to ensure their mutual inhibition, allowing Mmi1 to target Mei2 for ubiquitin-mediated downregulation, and in turn, allowing Mei2 accumulation to impede Mmi1 activity and fine-tune mitotic growth during meiotic mRNA degradation (161). The regulation mode between the above lncRNA and RBP is shown in Figure 2.

The limitation is that there were few extensive studies so far on the mechanism of lncRNA and RBP interaction in the field of atherosclerosis; most of the model construction in this review is based on studies on tumor-specific conditions.

## Conclusion and Perspective

Taken together, the studies discussed in this review show that lncRNAs and RBPs play biological functions in atherosclerotic ECs, SMCs, and macrophages; simultaneously, this review provided novel and understandable interaction models of lncRNAs and RBPs. Further extensive research is needed in the future to understand the functions and mechanisms of lncRNAs and RBPs, and provide novel and effective methods for the diagnosis, prevention and treatment of atherosclerosis.

## Author Contributions

YD drafted the manuscript. RY, SZ, and QX provided insightful discussions and comments on the manuscript. HZ, XP, and XZ discussed and revised the manuscript. All authors read and approved the final manuscript.

## Funding

This work was supported by the National Natural Science Foundation of China (No. 81771259) and the Natural Science Foundation of Shandong Province (ZR2020MH138).

## Conflict of Interest

The authors declare that the research was conducted in the absence of any commercial or financial relationships that could be construed as a potential conflict of interest.

## Publisher's Note

All claims expressed in this article are solely those of the authors and do not necessarily represent those of their affiliated organizations, or those of the publisher, the editors and the reviewers. Any product that may be evaluated in this article, or claim that may be made by its manufacturer, is not guaranteed or endorsed by the publisher.

## References

[1] Banerjee C Chimowitz MI. Stroke caused by atherosclerosis of the major intracranial arteries. Circul Res. (2017) 120:502–13. 10.1161/CIRCRESAHA.116.308441 28154100 PMC5312775

[2] Libby P Ridker PM Hansson GK. Progress and challenges in translating the biology of atherosclerosis. Nature. (2011) 473:317–25. 10.1038/nature10146 21593864

[3] Falk E. Pathogenesis of atherosclerosis. J Am Coll Cardiol. (2006) 47(8 Suppl):C7–12. 10.1016/j.jacc.2005.09.068 16631513

[4] Statello L Guo CJ Chen LL Huarte M. Gene regulation by long non-coding RNAs and its biological functions. Nat Rev Mol Cell Biol. (2021) 22:96–118. 10.1038/s41580-020-00315-9 33353982 PMC7754182

[5] Fasolo F Di Gregoli K Maegdefessel L Johnson JL. Non-coding RNAs in cardiovascular cell biology and atherosclerosis. Cardiovasc Res. (2019) 115:1732–56. 10.1093/cvr/cvz203 31389987 PMC7967706

[6] Shan K Jiang Q Wang XQ Wang YN Yang H Yao MD. Role of long non-coding RNA-RNCR3 in atherosclerosis-related vascular dysfunction. Cell Death Dis. (2016) 7:e2248. 10.1038/cddis.2016.145 27253412 PMC5143375

[7] Zhang S Wang J Qu MJ Wang K Ma AJ Pan XD. Novel insights into the potential diagnostic value of circulating exosomal IncRNA-related networks in large artery atherosclerotic stroke. Front Mol Biosci. (2021) 8:682769. 10.3389/fmolb.2021.682769 34095232 PMC8176956

[8] Xu S Kamato D Little PJ Nakagawa S Pelisek J Jin ZG. Targeting epigenetics and non-coding RNAs in atherosclerosis: from mechanisms to therapeutics. Pharmacol Ther. (2019) 196:15–43. 10.1016/j.pharmthera.2018.11.003 30439455 PMC6450782

[9] Ferrè F Colantoni A Helmer-Citterich M. Revealing protein-lncRNA interaction. Brief Bioinformatics. (2016) 17:106–16. 10.1093/bib/bbv031 26041786 PMC4719072

[10] Gerstberger S Hafner M Tuschl T. A census of human RNA-binding proteins. Nat Rev Genet. (2014) 15:829–45. 10.1038/nrg3813 25365966 PMC11148870

[11] Carlevaro-Fita J Johnson R. Global positioning system: understanding long noncoding RNAs through subcellular localization. Mol Cell. (2019) 73:869–83. 10.1016/j.molcel.2019.02.008 30849394

[12] Barton M Meyer MR. HuR-ry up: how hydrogen sulfide protects against atherosclerosis. Circulation. (2019) 139:115–8. 10.1161/CIRCULATIONAHA.118.036854 30592653

[13] Qian X Zhao J Yeung PY Zhang QC Kwok CK. Revealing lncRNA structures and interactions by sequencing-based approaches. Trends Biochem Sci. (2019) 44:33–52. 10.1016/j.tibs.2018.09.012 30459069

[14] Ma L Bajic VB Zhang Z. On the classification of long non-coding RNAs. RNA Biol. (2013) 10:925–33. 10.4161/rna.24604 23696037 PMC4111732

[15] Ali T Grote P. Beyond the RNA-dependent function of LncRNA genes. Elife. (2020) 9:e60583. 10.7554/eLife.60583 33095159 PMC7584451

[16] Kapusta A Kronenberg Z Lynch VJ Zhuo X Ramsay L Bourque G. Transposable elements are major contributors to the origin, diversification, and regulation of vertebrate long noncoding RNAs. PLoS Genet. (2013) 9:e1003470. 10.1371/journal.pgen.1003470 23637635 PMC3636048

[17] Cuevas-Diaz Duran R Wei H Kim DH Wu JQ. Invited review: long non-coding RNAs: important regulators in the development, function and disorders of the central nervous system. Neuropathol Appl Neurobiol. (2019) 45:538–56. 10.1111/nan.12541 30636336 PMC6626588

[18] Guo CJ Xu G Chen LL. Mechanisms of long noncoding RNA nuclear retention. Trends Biochem Sci. (2020) 45:947–60. 10.1016/j.tibs.2020.07.001 32800670

[19] Constanty F Shkumatava A. lncRNAs in development and differentiation: from sequence motifs to functional characterization. Development. (2021) 148:182741. 10.1242/dev.182741 33441380

[20] Marchese FP Raimondi I Huarte M. The multidimensional mechanisms of long noncoding RNA function. Genome Biol. (2017) 18:206. 10.1186/s13059-017-1348-2 29084573 PMC5663108

[21] Hentze MW Castello A Schwarzl T Preiss T. A brave new world of RNA-binding proteins. Nat Rev Mol Cell Biol. (2018) 19:327–41. 10.1038/nrm.2017.130 29339797

[22] Corley M Burns MC Yeo GW. How RNA-binding proteins interact with RNA: molecules and mechanisms. Mol Cell. (2020) 78:9–29. 10.1016/j.molcel.2020.03.011 32243832 PMC7202378

[23] Gebauer F Schwarzl T Valcárcel J Hentze MW. RNA-binding proteins in human genetic disease. Nat Rev Genet. (2021) 22:185–98. 10.1038/s41576-020-00302-y 33235359

[24] Siang DTC Lim YC Kyaw AMM Win KN Chia SY Degirmenci U. The RNA-binding protein HuR is a negative regulator in adipogenesis. Nat Commun. (2020) 11:213. 10.1038/s41467-019-14001-8 31924774 PMC6954112

[25] Broadbent HM Peden JF Lorkowski S Goel A Ongen H Green F. Susceptibility to coronary artery disease and diabetes is encoded by distinct, tightly linked SNPs in the ANRIL locus on chromosome 9p. Hum Mol Genet. (2008) 17:806–14. 10.1093/hmg/ddm352 18048406

[26] Congrains A Kamide K Katsuya T Yasuda O Oguro R Yamamoto K. CVD-associated non-coding RNA, ANRIL, modulates expression of atherogenic pathways in VSMC. Biochem Biophys Res Commun. (2012) 419:612–6. 10.1016/j.bbrc.2012.02.050 22382030

[27] Congrains A Kamide K Oguro R Yasuda O Miyata K Yamamoto E. Genetic variants at the 9p21 locus contribute to atherosclerosis through modulation of ANRIL and CDKN2A/B. Atherosclerosis. (2012) 220:449–55. 10.1016/j.atherosclerosis.2011.11.017 22178423

[28] Motterle A Pu X Wood H Xiao Q Gor S Ng FL. Functional analyses of coronary artery disease associated variation on chromosome 9p21 in vascular smooth muscle cells. Hum Mol Genet. (2012) 21:4021–9. 10.1093/hmg/dds224 22706276 PMC3428153

[29] Holdt LM Hoffmann S Sass K Langenberger D Scholz M Krohn K. Alu elements in ANRIL non-coding RNA at chromosome 9p21 modulate atherogenic cell functions through trans-regulation of gene networks. PLoS Genet. (2013) 9:e1003588. 10.1371/journal.pgen.1003588 23861667 PMC3701717

[30] Huang T Zhao HY Zhang XB Gao XL Peng WP Zhou Y. LncRNA ANRIL regulates cell proliferation and migration via sponging miR-339-5p and regulating FRS2 expression in atherosclerosis. Eur Rev Med Pharmacol Sci. (2020) 24:1956–69. 10.26355/eurrev_202002_20373 32141564

[31] Hu YW Yang JY Ma X Chen ZP Hu YR Zhao JY. A lincRNA-DYNLRB2-2/GPR119/GLP-1R/ABCA1-dependent signal transduction pathway is essential for the regulation of cholesterol homeostasis. J Lipid Res. (2014) 55:681–97. 10.1194/jlr.M044669 24493833 PMC3966702

[32] Wu G Cai J Han Y Chen J Huang ZP Chen C. LincRNA-p21 regulates neointima formation, vascular smooth muscle cell proliferation, apoptosis, and atherosclerosis by enhancing p53 activity. Circulation. (2014) 130:1452–65. 10.1161/CIRCULATIONAHA.114.011675 25156994 PMC4244705

[33] Hu YW Zhao JY Li SF Huang JL Qiu YR Ma X. RP5-833A20.1/miR-382-5p/NFIA-dependent signal transduction pathway contributes to the regulation of cholesterol homeostasis and inflammatory reaction. Arterioscler Thromb Vasc Biol. (2015) 35:87–101. 10.1161/ATVBAHA.114.304296 25265644

[34] Huang C Hu YW Zhao JJ Ma X Zhang Y Guo FX. Long noncoding RNA HOXC-AS1 suppresses Ox-LDL-induced cholesterol accumulation through promoting HOXC6 expression in THP-1 macrophages. DNA Cell Biol. (2016) 35:722–9. 10.1089/dna.2016.3422 27574949

[35] Ma Y Huang D Yang F Tian M Wang Y Shen D. Long noncoding RNA highly upregulated in liver cancer regulates the tumor necrosis factor-α-induced apoptosis in human vascular endothelial cells. DNA Cell Biol. (2016) 35:296–300. 10.1089/dna.2015.3203 26981838

[36] Peng Y Meng K Jiang L Zhong Y Yang Y Lan Y. Thymic stromal lymphopoietin-induced HOTAIR activation promotes endothelial cell proliferation and migration in atherosclerosis. Biosci Rep. (2017) 37:BSR20170351. 10.1042/BSR20170351 28615347 PMC5518535

[37] Liu J Huang GQ Ke ZP. Silence of long intergenic noncoding RNA HOTAIR ameliorates oxidative stress and inflammation response in ox-LDL-treated human macrophages by upregulating miR-330-5p. J Cell Physiol. (2019) 234:5134–42. 10.1002/jcp.27317 30187491

[38] Zhang DD Wang WT Xiong J Xie XM Cui SS Zhao ZG. Long noncoding RNA LINC00305 promotes inflammation by activating the AHRR-NF-κB pathway in human monocytes. Sci Rep. (2017) 7:46204. 10.1038/srep46204 28393844 PMC5385552

[39] Li FP Lin DQ Gao LY. LncRNA TUG1 promotes proliferation of vascular smooth muscle cell and atherosclerosis through regulating miRNA-21/PTEN axis. Eur Rev Med Pharmacol Sci. (2018) 22:7439–47. 10.26355/eurrev_201811_16284 30468492

[40] Tang Y Hu J Zhong Z Liu Y Wang Y. Long noncoding RNA TUG1 promotes the function in ox-LDL-Treated HA-VSMCs via miR-141-3p/ROR2 Axis. Cardiovasc Ther. (2020) 2020:6758934. 10.1155/2020/6758934 32565910 PMC7285414

[41] Li S Sun Y Zhong L Xiao Z Yang M Chen M. The suppression of ox-LDL-induced inflammatory cytokine release and apoptosis of HCAECs by long non-coding RNA-MALAT1 via regulating microRNA-155/SOCS1 pathway. Nutr Metab Cardiovasc Dis. (2018) 28:1175–87. 10.1016/j.numecd.2018.06.017 30314869

[42] Li H Zhao Q Chang L Wei C Bei H Yin Y. LncRNA MALAT1 modulates ox-LDL induced EndMT through the Wnt/beta-catenin signaling pathway. Lipids Health Dis. (2019) 18:62. 10.1186/s12944-019-1006-7 30871555 PMC6417088

[43] Li Y Shen S Ding S Wang L. LincRNA DYN-LRB2-2 upregulates cholesterol efflux by decreasing TLR2 expression in macrophages. J Cell Biochem. (2018) 119:1911–21. 10.1002/jcb.26352 28815701

[44] Miao C Cao H Zhang Y Guo X Wang Z Wang J. LncRNA DIGIT accelerates tube formation of vascular endothelial cells by sponging miR-134. Int Heart J. (2018) 59:1086–95. 10.1536/ihj.17-290 30158376

[45] Sallam T Jones M Thomas BJ Wu X Gilliland T Qian K. Transcriptional regulation of macrophage cholesterol efflux and atherogenesis by a long noncoding RNA. Nat Med. (2018) 24: 304–12. 10.1038/nm.4479 29431742 PMC5839972

[46] Tian S Yuan Y Li Z Gao M Lu Y Gao H. LncRNA UCA1 sponges miR-26a to regulate the migration and proliferation of vascular smooth muscle cells. Gene. (2018) 673:159–66. 10.1016/j.gene.2018.06.031 29908280

[47] Xu X Ma C Liu C Duan Z Zhang L. Knockdown of long noncoding RNA XIST alleviates oxidative low-density lipoprotein-mediated endothelial cells injury through modulation of miR-320/NOD2 axis. Biochem Biophys Res Commun. (2018) 503:586–92. 10.1016/j.bbrc.2018.06.042 29902461

[48] Yang S Sun J. LncRNA SRA deregulation contributes to the development of atherosclerosis by causing dysfunction of endothelial cells through repressing the expression of adipose triglyceride lipase. Mol Med Rep. (2018) 18:5207–14. 10.3892/mmr.2018.9497 30272285

[49] Yao X Yan C Zhang L Li Y Wan Q. LncRNA ENST00113 promotes proliferation, survival, and migration by activating PI3K/Akt/mTOR signaling pathway in atherosclerosis. Medicine. (2018) 97:e0473. 10.1097/MD.0000000000010473 29668625 PMC5916647

[50] Ye J Wang C Wang D Yuan H. LncRBA GSA5, up-regulated by ox-LDL, aggravates inflammatory response and MMP expression in THP-1 macrophages by acting like a sponge for miR-221. Exp Cell Res. (2018) 369:348–55. 10.1016/j.yexcr.2018.05.039 29859752

[51] Liang W Fan T Liu L Zhang L. Knockdown of growth-arrest specific transcript 5 restores oxidized low-density lipoprotein-induced impaired autophagy flux via upregulating miR-26a in human endothelial cells. Eur J Pharmacol. (2019) 843:154–61. 10.1016/j.ejphar.2018.11.005 30468731

[52] Chen L Yang W Guo Y Chen W Zheng P Zeng J. Exosomal lncRNA GAS5 regulates the apoptosis of macrophages and vascular endothelial cells in atherosclerosis. PLoS ONE. (2017) 12:e0185406. 10.1371/journal.pone.0185406 28945793 PMC5612752

[53] Zhang L Cheng H Yue Y Li S Zhang D He R. H19 knockdown suppresses proliferation and induces apoptosis by regulating miR-148b/WNT/beta-catenin in ox-LDL -stimulated vascular smooth muscle cells. J Biomed Sci. (2018) 25:11. 10.1186/s12929-018-0418-4 29415742 PMC5804091

[54] Zhang Y Liu X Bai X Lin Y Li Z Fu J. Melatonin prevents endothelial cell pyroptosis via regulation of long noncoding RNA MEG3/miR-223/NLRP3 axis. J Pineal Res. (2018) 64:e12449. 10.1111/jpi.12449 29024030

[55] Bai Y Zhang Q Su Y Pu Z Li K. Modulation of the proliferation/apoptosis balance of vascular smooth muscle cells in atherosclerosis by lncRNA-MEG3 via regulation of miR-26a/Smad1 Axis. Int Heart J. (2019) 60:444–50. 10.1536/ihj.18-195 30745534

[56] Zhong X Ma X Zhang L Li Y Li Y He R. MIAT promotes proliferation and hinders apoptosis by modulating miR-181b/STAT3 axis in ox-LDL-induced atherosclerosis cell models. Biomed Pharmacother. (2018) 97:1078–85. 10.1016/j.biopha.2017.11.052 29136944

[57] Sun G Li Y Ji Z. Up-regulation of MIAT aggravates the atherosclerotic damage in atherosclerosis mice through the activation of PI3K/Akt signaling pathway. Drug Deliv. (2019) 26:641–9. 10.1080/10717544.2019.1628116 31237148 PMC6598488

[58] An JH Chen ZY Ma QL Wang HJ Zhang JQ Shi FW. LncRNA SNHG16 promoted proliferation and inflammatory response of macrophages through miR-17-5p/NF-kappaB signaling pathway in patients with atherosclerosis. Eur Rev Med Pharmacol Sci. (2019) 23:8665–77. 10.26355/eurrev_201910_19184 31646601

[59] Lin Y Tian G Zhang H Yuan W Xie Y Yang Y. Long non-coding RNA SNHG16 regulates human aortic smooth muscle cell proliferation and migration via sponging miR-205 and modulating Smad2. J Cell Mol Med. (2019) 23:6919–29. 10.1111/jcmm.14576 31441592 PMC6787464

[60] Cui C Wang X Shang XM Li L Ma Y Zhao GY. lncRNA 430945 promotes the proliferation and migration of vascular smooth muscle cells via the ROR2/RhoA signaling pathway in atherosclerosis. Mol Med Rep. (2019) 19:4663–72. 10.3892/mmr.2019.10137 30957191 PMC6522828

[61] Guo FX Wu Q Li P Zheng L Ye S Dai XY. The role of the LncRNA-FA2H-2-MLKL pathway in atherosclerosis by regulation of autophagy flux and inflammation through mTOR-dependent signaling. Cell Death Differ. (2019) 26:1670–87. 10.1038/s41418-018-0235-z 30683918 PMC6748100

[62] Hu YW Guo FX Xu YJ Li P Lu ZF McVey DG. Long noncoding RNA NEXN-AS1 mitigates atherosclerosis by regulating the actin-binding protein NEXN. J Clin Invest. (2019) 129:1115–28. 10.1172/JCI98230 30589415 PMC6391138

[63] Khyzha N Khor M DiStefano PV Wang L Matic L Hedin U. Regulation of CCL2 expression in human vascular endothelial cells by a neighboring divergently transcribed long noncoding RNA. Proc Natl Acad Sci USA. (2019) 116:16410–9. 10.1073/pnas.1904108116 31350345 PMC6697820

[64] Li H Han S Sun Q Yao Y Li S Yuan C. Long non-coding RNA CDKN2B-AS1 reduces inflammatory response and promotes cholesterol efflux in atherosclerosis by inhibiting ADAM10 expression. Aging. (2019) 11:1695–715. 10.18632/aging.101863 30926762 PMC6461186

[65] Liu Y Chen Y Tan L Zhao H Xiao N. Linc00299/miR-490-3p/AURKA axis regulates cell growth and migration in atherosclerosis. Heart Vessels. (2019) 34:1370–80. 10.1007/s00380-019-01356-7 30734057

[66] Lu Q Meng Q Qi M Li F Liu B. Shear-sensitive lncRNA AF131217.1 Inhibits Inflammation in HUVECs via regulation of KLF4. Hypertension. (2019) 73:e25–34. 10.1161/HYPERTENSIONAHA.118.12476 30905197

[67] Song A Feng R Gao J Yang C. Long noncoding RNA Alu-mediated p21 transcriptional regulator promotes proliferation, migration, and pipe-formation of human microvascular endothelial cells by sponging miR-126. J Cell Biochem. (2019) 120:19858–67. 10.1002/jcb.29291 31310378

[68] Sun C Fu Y Gu X Xi X Peng X Wang C. Macrophage-enriched lncRNA RAPIA: a novel therapeutic target for atherosclerosis. Arterioscler Thromb Vasc Biol. (2020) 40:1464–78. 10.1161/ATVBAHA.119.313749 32268789

[69] Sun Y Zhao JT Chi BJ Wang KF. Long noncoding RNA SNHG12 promotes vascular smooth muscle cell proliferation and migration via regulating miR-199a-5p/HIF-1alpha. Cell Biol Int. (2020) 44:1714–26. 10.1002/cbin.11365 32339345

[70] Tao K Hu Z Zhang Y Jiang D Cheng H. LncRNA CASC11 improves atherosclerosis by downregulating IL-9 and regulating vascular smooth muscle cell apoptosis and proliferation. Biosci Biotechnol Biochem. (2019) 83:1284–8. 10.1080/09168451.2019.1597621 30915898

[71] Wang L Xia JW Ke ZP Zhang BH. Blockade of NEAT1 represses inflammation response and lipid uptake via modulating miR-342-3p in human macrophages THP-1 cells. J Cell Physiol. (2019) 234:5319–26. 10.1002/jcp.27340 30259979

[72] Zhang B Dong Y Zhao Z. LncRNA MEG8 regulates vascular smooth muscle cell proliferation, migration and apoptosis by targeting PPARalpha. Biochem Biophys Res Commun. (2019) 510:171–6. 10.1016/j.bbrc.2019.01.074 30670309

[73] Zhang L Zhou C Qin Q Liu Z Li P. LncRNA LEF1-AS1 regulates the migration and proliferation of vascular smooth muscle cells by targeting miR-544a/PTEN axis. J Cell Biochem. (2019) 120:14670–8. 10.1002/jcb.28728 31016789

[74] Zhen Z Ren S Ji H Ding X Zou P Lu J. The lncRNA DAPK-IT1 regulates cholesterol metabolism and inflammatory response in macrophages and promotes atherogenesis. Biochem Biophys Res Commun. (2019) 516 1234–41. 10.1016/j.bbrc.2019.06.113 31300197

[75] Zhu X Chen D Liu Y Yu J Qiao L Lin S. Long noncoding RNA HOXA-AS3 integrates NF-kappaB signaling to regulate endothelium inflammation. Mol Cell Biol. (2019) 39:e00139–19. 10.1128/MCB.00139-19 31285272 PMC6751633

[76] Bian W Jing X Yang Z Shi Z Chen R Xu A. Downregulation of LncRNA NORAD promotes Ox-LDL-induced vascular endothelial cell injury and atherosclerosis. Aging. (2020) 12:6385–400. 10.18632/aging.103034 32267831 PMC7185106

[77] Cheng Q Zhang M Zhang M Ning L Chen D. Long non-coding RNA LOC285194 regulates vascular smooth muscle cell apoptosis in atherosclerosis. Bioengineered. (2020) 11:53–60. 10.1080/21655979.2019.1705054 31884873 PMC6961585

[78] Guo X Liu Y Zheng X Han Y Cheng J. HOTTIP knockdown inhibits cell proliferation and migration via regulating miR-490-3p/HMGB1 axis and PI3K-AKT signaling pathway in ox-LDL-induced VSMCs. Life Sci. (2020) 248:117445. 10.1016/j.lfs.2020.117445 32081664

[79] Ji Z Chi J Sun H Ru A Ni T Zhang J. Linc-ROR targets FGF2 to regulate HASMC proliferation and migration via sponging miR-195-5p. Gene. (2020) 725:144143. 10.1016/j.gene.2019.144143 31629816

[80] Liu Y Cui X Wang C Zhao S. LncRNA HCG11 regulates proliferation and apoptosis of vascular smooth muscle cell through targeting miR-144-3p/FOXF1 axis in atherosclerosis. Biol Res. (2020) 53:44. 10.1186/s40659-020-00306-2 33008472 PMC7532112

[81] Simion V Zhou H Haemmig S Pierce JB Mendes S Tesmenitsky Y. A macrophage-specific lncRNA regulates apoptosis and atherosclerosis by tethering HuR in the nucleus. Nat Commun. (2020) 11:6135. 10.1038/s41467-020-19664-2 33262333 PMC7708640

[82] Wang Q Yang Y Fu X Wang Z Liu Y Li M. Long noncoding RNA XXYLT1-AS2 regulates proliferation and adhesion by targeting the RNA binding protein FUS in HUVEC. Atherosclerosis. (2020) 298:58–69. 10.1016/j.atherosclerosis.2020.02.018 32171981

[83] Wang Y Zhang CX Ge SL Gong WH. CTBP1AS2 inhibits proliferation and induces autophagy in oxLDLstimulated vascular smooth muscle cells by regulating miR1955p/ATG14. Int J Mol Med. (2020) 46:839–48. 10.3892/ijmm.2020.4624 32626936

[84] Wang YQ Xu ZM Wang XL Zheng JK Du Q Yang JX. LncRNA FOXC2-AS1 regulated proliferation and apoptosis of vascular smooth muscle cell through targeting miR-1253/FOXF1 axis in atherosclerosis. Eur Rev Med Pharmacol Sci. (2020) 24:3302–14. 10.26355/eurrev_202003_20698 32271448

[85] Wu H Liu T Hou H. Knockdown of LINC00657 inhibits ox-LDL-induced endothelial cell injury by regulating miR-30c-5p/Wnt7b/beta-catenin. Mol Cell Biochem. (2020) 472:145–55. 10.1007/s11010-020-03793-9 32577947

[86] Ye F Zhang J Zhang Q Zhang J Chen C. Preliminary study on the mechanism of long noncoding RNA SENCR regulating the proliferation and migration of vascular smooth muscle cells. J Cell Physiol. (2020) 235:9635–43. 10.1002/jcp.29775 32401347

[87] Yu XH Deng WY Chen JJ Xu XD Liu XX Chen L. LncRNA kcnq1ot1 promotes lipid accumulation and accelerates atherosclerosis via functioning as a ceRNA through the miR-452-3p/HDAC3/ABCA1 axis. Cell Death Dis. (2020) 11:1043. 10.1038/s41419-020-03263-6 33293505 PMC7723992

[88] He X Lian Z Yang Y Wang Z Fu X Liu Y. Long Non-coding RNA PEBP1P2 suppresses proliferative VSMCs phenotypic switching and proliferation in atherosclerosis. Mol Ther Nucleic Acids. (2020) 22:84–98. 10.1016/j.omtn.2020.08.013 32916601 PMC7490454

[89] Zhang S Zhu X Li G. E2F1/SNHG7/miR-186-5p/MMP2 axis modulates the proliferation and migration of vascular endothelial cell in atherosclerosis. Life Sci. (2020) 257:118013. 10.1016/j.lfs.2020.118013 32603818

[90] Dong XH Lu ZF Kang CM Li XH Haworth KE Ma X. The long noncoding RNA RP11-728F11.4 promotes atherosclerosis. Arterioscler Thromb Vasc Biol. (2021) 41:1191–204. 10.1161/ATVBAHA.120.315114 33406853

[91] Mahmoud AD Ballantyne MD Miscianinov V Pinel K Hung J Scanlon JP. The human-specific and smooth muscle cell-enriched LncRNA SMILR promotes proliferation by regulating mitotic CENPF mRNA and drives cell-cycle progression which can be targeted to limit vascular remodeling. Circul Res. (2019) 125:535–51. 10.1161/CIRCRESAHA.119.314876 31339449 PMC6693924

[92] Mobin MB Gerstberger S Teupser D Campana B Charisse K Heim MH. The RNA-binding protein vigilin regulates VLDL secretion through modulation of Apob mRNA translation. Nat Commun. (2016) 7:12848. 10.1038/ncomms12848 27665711 PMC5052685

[93] Ramírez CM Lin CS Abdelmohsen K Goedeke L Yoon JH Madrigal-Matute J. RNA binding protein HuR regulates the expression of ABCA1. J Lipid Res. (2014) 55:1066–76. 10.1194/jlr.M044925 24729624 PMC4031938

[94] Yang C Eleftheriadou M Kelaini S Morrison T González MV Caines R. Targeting QKI-7 *in vivo* restores endothelial cell function in diabetes. Nat Commun. (2020) 11:3812. 10.1038/s41467-020-17468-y 32732889 PMC7393072

[95] Santovito D Egea V Bidzhekov K Natarelli L Mourão A Blanchet X. Autophagy unleashes noncanonical microRNA functions. Autophagy. (2020) 16:2294–6. 10.1080/15548627.2020.1830523 33054575 PMC7751630

[96] Nguyen MA Karunakaran D Geoffrion M Cheng HS Tandoc K Perisic Matic L. Extracellular vesicles secreted by atherogenic macrophages transfer microRNA to inhibit cell migration. Arterioscler Thromb Vasc Biol. (2018) 38:49–63. 10.1161/ATVBAHA.117.309795 28882869 PMC5884694

[97] Blackstock CD Higashi Y Sukhanov S Shai SY Stefanovic B Tabony AM. Insulin-like growth factor-1 increases synthesis of collagen type I via induction of the mRNA-binding protein LARP6 expression and binding to the 5' stem-loop of COL1a1 and COL1a2 mRNA. J Biol Chem. (2014) 289:7264–74. 10.1074/jbc.M113.518951 24469459 PMC3953245

[98] Jiang H Zhang J Du Y Jia X Yang F Si S. microRNA-185 modulates low density lipoprotein receptor expression as a key posttranscriptional regulator. Atherosclerosis. (2015) 243:523–32. 10.1016/j.atherosclerosis.2015.10.026 26523989

[99] Zhang H Taylor WR Joseph G Caracciolo V Gonzales DM Sidell N. mRNA-binding protein ZFP36 is expressed in atherosclerotic lesions and reduces inflammation in aortic endothelial cells. Arterioscler Thromb Vasc Biol. (2013) 33:1212–20. 10.1161/ATVBAHA.113.301496 23559629 PMC3844532

[100] Chen X He Y Zhu Y Du J Sun H. linc-AAM facilitates gene expression contributing to macrophage activation and adaptive immune responses. Cell Rep. (2021) 34:108584. 10.1016/j.celrep.2020.108584 33406422

[101] Haneklaus M O'Neil JD Clark AR Masters SL O'Neill LAJ. The RNA-binding protein Tristetraprolin (TTP) is a critical negative regulator of the NLRP3 inflammasome. J Biol Chem. (2017) 292:6869–81. 10.1074/jbc.M116.772947 28302726 PMC5409458

[102] Kruger-Genge A Blocki A Franke RP Jung F. Vascular endothelial cell biology: an update. Int J Mol Sci. (2019) 20:4411. 10.3390/ijms20184411 31500313 PMC6769656

[103] Eelen G de Zeeuw P Treps L Harjes U Wong BW Carmeliet P. Endothelial cell metabolism. Physiol Rev. (2018) 98:3–58. 10.1152/physrev.00001.2017 29167330 PMC5866357

[104] Singh KK Matkar PN Pan Y Quan A Gupta V Teoh H. Endothelial long non-coding RNAs regulated by oxidized LDL. Mol Cell Biochem. (2017) 431:139–49. 10.1007/s11010-017-2984-2 28316063

[105] Wang J Chen L Li H Yang J Gong Z Wang B. Clopidogrel reduces apoptosis and promotes proliferation of human vascular endothelial cells induced by palmitic acid via suppression of the long non-coding RNA HIF1A-AS1 *in vitro*. Mol Cell Biochem. (2015) 404:203–10. 10.1007/s11010-015-2379-1 25761653

[106] Hennessy EJ Parker AE O'Neill LA. Targeting toll-like receptors: emerging therapeutics? Nat Rev Drug Discov. (2010) 9:293–307. 10.1038/nrd3203 20380038

[107] Michelsen KS Wong MH Shah PK Zhang W Yano J Doherty TM. Lack of Toll-like receptor 4 or myeloid differentiation factor 88 reduces atherosclerosis and alters plaque phenotype in mice deficient in apolipoprotein E. Proc Natl Acad Sci USA. (2004) 101:10679–84. 10.1073/pnas.0403249101 15249654 PMC489994

[108] Ohtsuka T Nakanishi H Ikeda W Satoh A Momose Y Nishioka H. Nexilin: a novel actin filament-binding protein localized at cell-matrix adherens junction. J Cell Biol. (1998) 143:1227–38. 10.1083/jcb.143.5.1227 9832551 PMC2133087

[109] Moore KJ Sheedy FJ Fisher EA. Macrophages in atherosclerosis: a dynamic balance. Nat Rev Immunol. (2013) 13:709–21. 10.1038/nri3520 23995626 PMC4357520

[110] Tong Y Cai L Yang S Liu S Wang Z Cheng J. The research progress of vascular macrophages and atherosclerosis. Oxid Med Cell Longev. (2020) 2020:7308736. 10.1155/2020/7308736 32566098 PMC7267869

[111] Tabas I Lichtman AH. Monocyte-macrophages and T Cells in atherosclerosis. Immunity. (2017) 47:621–34. 10.1016/j.immuni.2017.09.008 29045897 PMC5747297

[112] Schultze JL Schmieder A Goerdt S. Macrophage activation in human diseases. Semin Immunol. (2015) 27:249–56. 10.1016/j.smim.2015.07.003 26303100

[113] Shapouri-Moghaddam A Mohammadian S Vazini H Taghadosi M Esmaeili SA Mardani F. Macrophage plasticity, polarization, and function in health and disease. J Cell Physiol. (2018) 233:6425–40. 10.1002/jcp.26429 29319160 PMC13482560

[114] Jinnouchi H Guo L Sakamoto A Torii S Sato Y Cornelissen A. Diversity of macrophage phenotypes and responses in atherosclerosis. Cell Mol Life Sci. (2020) 77:1919–32. 10.1007/s00018-019-03371-3 31720740 PMC11104939

[115] Gordon S Taylor PR. Monocyte and macrophage heterogeneity. Nat Rev Immunol. (2005) 5:953–64. 10.1038/nri1733 16322748

[116] Guilliams M Mildner A Yona S. Developmental and functional heterogeneity of monocytes. Immunity. (2018) 49:595–613. 10.1016/j.immuni.2018.10.005 30332628

[117] Vergadi E Ieronymaki E Lyroni K Vaporidi K Tsatsanis C. Akt signaling pathway in macrophage activation and M1/M2 polarization. J Immunol. (2017) 198:1006–14. 10.4049/jimmunol.1601515 28115590

[118] Lin Y Zhao JL Zheng QJ Jiang X Tian J Liang SQ. Notch signaling modulates macrophage polarization and phagocytosis through direct suppression of signal regulatory protein α expression. Front Immunol. (2018) 9:1744. 10.3389/fimmu.2018.01744 30105024 PMC6077186

[119] Qin H Holdbrooks AT Liu Y Reynolds SL Yanagisawa LL Benveniste EN. SOCS3 deficiency promotes M1 macrophage polarization and inflammation. J Immunol. (2012) 189:3439–48. 10.4049/jimmunol.1201168 22925925 PMC4184888

[120] O'Shea JJ Pesu M Borie DC Changelian PS. A new modality for immunosuppression: targeting the JAK/STAT pathway. Nat Rev Drug Discov. (2004) 3:555–64. 10.1038/nrd1441 15232577

[121] Zhang J Shi H Zhang N Hu L Jing W Pan J. Interleukin-4-loaded hydrogel scaffold regulates macrophages polarization to promote bone mesenchymal stem cells osteogenic differentiation via TGF-β1/Smad pathway for repair of bone defect. Cell Prolif. (2020) 53:e12907. 10.1111/cpr.12907 32951298 PMC7574882

[122] Abaricia JO Shah AH Chaubal M Hotchkiss KM Olivares-Navarrete R. Wnt signaling modulates macrophage polarization and is regulated by biomaterial surface properties. Biomaterials. (2020) 243:119920. 10.1016/j.biomaterials.2020.119920 32179303 PMC7191325

[123] Han Y Qiu H Pei X Fan Y Tian H Geng J. Low-dose sinapic acid abates the pyroptosis of macrophages by downregulation of lncRNA-MALAT1 in rats with diabetic atherosclerosis. J Cardiovasc Pharmacol. (2018) 71:104–12. 10.1097/FJC.0000000000000550 29095793

[124] Wells ML Perera L Blackshear PJ. An ancient family of RNA-binding proteins: still important! Trends Biochem Sci. (2017) 42:285–96. 10.1016/j.tibs.2016.12.003 28096055 PMC5376222

[125] Patial S Blackshear PJ. Tristetraprolin as a therapeutic target in inflammatory disease. Trends Pharmacol Sci. (2016) 37:811–21. 10.1016/j.tips.2016.07.002 27503556 PMC5030171

[126] Lai WS Wells ML Perera L Blackshear PJ. The tandem zinc finger RNA binding domain of members of the tristetraprolin protein family. Wiley Interdiscip Rev RNA. (2019) 10:e1531. 10.1002/wrna.1531 30864256 PMC6570553

[127] Saini Y Chen J Patial S. The tristetraprolin family of RNA-binding proteins in cancer: progress and future prospects. Cancers. (2020) 12:1539. 10.3390/cancers12061539 32545247 PMC7352335

[128] Jonas K Calin GA Pichler M. RNA-Binding proteins as important regulators of long non-coding RNAs in cancer. Int J Mol Sci. (2020) 21:2969. 10.3390/ijms21082969 32340118 PMC7215867

[129] Basatemur GL Jørgensen HF Clarke MCH Bennett MR Mallat Z. Vascular smooth muscle cells in atherosclerosis. Nat Rev Cardiol. (2019) 16:727–44. 10.1038/s41569-019-0227-9 31243391

[130] Hu D Yin C Luo S Habenicht AJR Mohanta SK. Vascular smooth muscle cells contribute to atherosclerosis immunity. Front Immunol. (2019) 10:1101. 10.3389/fimmu.2019.01101 31164888 PMC6534067

[131] Grootaert MOJ Moulis M Roth L Martinet W Vindis C Bennett MR. Vascular smooth muscle cell death, autophagy and senescence in atherosclerosis. Cardiovasc Res. (2018) 114:622–34. 10.1093/cvr/cvy007 29360955

[132] Perrotta I. The use of electron microscopy for the detection of autophagy in human atherosclerosis. Micron. (2013) 50:7–13. 10.1016/j.micron.2013.03.007 23623713

[133] Allahverdian S Chaabane C Boukais K Francis GA Bochaton-Piallat ML. Smooth muscle cell fate and plasticity in atherosclerosis. Cardiovasc Res. (2018) 114:540–50. 10.1093/cvr/cvy022 29385543 PMC5852505

[134] Park E Maquat LE. Staufen-mediated mRNA decay. Wiley Interdiscip Rev RNA. (2013) 4:423–35. 10.1002/wrna.1168 23681777 PMC3711692

[135] Sun Q Tripathi V Yoon JH Singh DK Hao Q Min KW. MIR100 host gene-encoded lncRNAs regulate cell cycle by modulating the interaction between HuR and its target mRNAs. Nucleic Acids Res. (2018) 46:10405–16. 10.1093/nar/gky696 30102375 PMC6212728

[136] Abdelmohsen K Lal A Kim HH Gorospe M. Posttranscriptional orchestration of an anti-apoptotic program by HuR. Cell Cycle. (2007) 6:1288–92. 10.4161/cc.6.11.4299 17534146

[137] Ray M Gabunia K Vrakas CN Herman AB Kako F Kelemen SE. Genetic deletion of IL-19 (Interleukin-19) exacerbates atherogenesis in Il19(-/-) × Ldlr(-/-) double knockout mice by dysregulation of mRNA stability protein HuR (Human Antigen R). Arterioscler Thromb Vasc Biol. (2018) 38:1297–308. 10.1161/ATVBAHA.118.310929 29674474 PMC5970062

[138] Liu S Jiang X Cui X Wang J Liu S Li H. Smooth muscle-specific HuR knockout induces defective autophagy and atherosclerosis. Cell Death Dis. (2021) 12:385. 10.1038/s41419-021-03671-2 33837179 PMC8035143

[139] Fu X Zhai S Yuan J. Endothelial HuR deletion reduces the expression of proatherogenic molecules and attenuates atherosclerosis. Int Immunopharmacol. (2018) 65:248–55. 10.1016/j.intimp.2018.09.023 30340104

[140] Geuens T Bouhy D Timmerman V. The hnRNP family: insights into their role in health and disease. Hum Genet. (2016) 135:851–67. 10.1007/s00439-016-1683-5 27215579 PMC4947485

[141] Sun X Haider Ali MSS Moran M. The role of interactions of long non-coding RNAs and heterogeneous nuclear ribonucleoproteins in regulating cellular functions. Biochem J. (2017) 474:2925–35. 10.1042/BCJ20170280 28801479 PMC5553131

[142] Fabbiano F Corsi J Gurrieri E Trevisan C Notarangelo M D'Agostino VG. RNA packaging into extracellular vesicles: an orchestra of RNA-binding proteins? J Extracell Vesicles. (2020) 10:e12043. 10.1002/jev2.12043 33391635 PMC7769857

[143] Laury-Kleintop LD Tresini M Hammond O. Compartmentalization of hnRNP-K during cell cycle progression and its interaction with calponin in the cytoplasm. J Cell Biochem. (2005) 95:1042–56. 10.1002/jcb.20486 15962305

[144] Panchenko MP Silva N Stone JR. Up-regulation of a hydrogen peroxide-responsive pre-mRNA binding protein in atherosclerosis and intimal hyperplasia. Cardiovasc Pathol. (2009) 18:167–72. 10.1016/j.carpath.2008.03.008 18508286 PMC2723736

[145] Brooke GN Culley RL Dart DA Mann DJ Gaughan L McCracken SR. FUS/TLS is a novel mediator of androgen-dependent cell-cycle progression and prostate cancer growth. Cancer Res. (2011) 71:914–24. 10.1158/0008-5472.CAN-10-0874 21169411

[146] Lee J Nguyen PT Shim HS Hyeon SJ Im H Choi MH. EWSR1, a multifunctional protein, regulates cellular function and aging via genetic and epigenetic pathways. Biochim Biophys Acta Mol Basis Dis. (2019) 1865:1938–45. 10.1016/j.bbadis.2018.10.042 30481590 PMC6527469

[147] Rodrigues AC Adamoski D Genelhould G Zhen F Yamaguto GE Araujo-Souza PS. NEAT1 and MALAT1 are highly expressed in saliva and nasopharyngeal swab samples of COVID-19 patients. Mol Oral Microbiol. (2021). 10.1111/omi.12351. [Epub ahead of print]. 34463043 PMC8661855

[148] Marin-Bejar O Mas AM Gonzalez J Martinez D Athie A Morales X. The human lncRNA LINC-PINT inhibits tumor cell invasion through a highly conserved sequence element. Genome Biol. (2017) 18:202. 10.1186/s13059-017-1331-y 29078818 PMC5660458

[149] Chen HS Tong HS Zhao Y Hong CY Bin JP Su L. Differential expression pattern of exosome long non-coding RNAs (lncRNAs) and MicroRNAs (miRNAs) in vascular endothelial cells under heat stroke. Med Sci Monit. (2018) 24:7965–74. 10.12659/MSM.909983 30399613 PMC6234752

[150] Beaulieu YB Kleinman CL Landry-Voyer AM Majewski J Bachand F. Polyadenylation-dependent control of long noncoding RNA expression by the poly(A)-binding protein nuclear 1. PLoS Genet. (2012) 8:e1003078. 10.1371/journal.pgen.1003078 23166521 PMC3499365

[151] Zhou X Li X Yu L Wang R Hua D Shi C. The RNA-binding protein SRSF1 is a key cell cycle regulator via stabilizing NEAT1 in glioma. Int J Biochem Cell Biol. (2019) 113:75–86. 10.1016/j.biocel.2019.06.003 31200124

[152] Chai Y Liu J Zhang Z Liu L. HuR-regulated lncRNA NEAT1 stability in tumorigenesis and progression of ovarian cancer. Cancer Med. (2016) 5:1588–98. 10.1002/cam4.710 27075229 PMC4944886

[153] Takayama K Horie-Inoue K Katayama S Suzuki T Tsutsumi S Ikeda K. Androgen-responsive long noncoding RNA CTBP1-AS promotes prostate cancer. EMBO J. (2013) 32:1665–80. 10.1038/emboj.2013.99 23644382 PMC3680743

[154] Tian FJ He XY Wang J Li X Ma XL Wu F. Elevated tristetraprolin impairs trophoblast invasion in women with recurrent miscarriage by destabilization of HOTAIR. Mol Ther Nucleic Acids. (2018) 12:600–9. 10.1016/j.omtn.2018.07.001 30195796 PMC6078837

[155] Chen C Luo Y He W Zhao Y Kong Y Liu H. Exosomal long noncoding RNA LNMAT2 promotes lymphatic metastasis in bladder cancer. J Clin Invest. (2020) 130:404–21. 10.1172/JCI130892 31593555 PMC6934220

[156] Han M Gu Y Lu P Li J Cao H Li X. Exosome-mediated lncRNA AFAP1-AS1 promotes trastuzumab resistance through binding with AUF1 and activating ERBB2 translation. Mol Cancer. (2020) 19:26. 10.1186/s12943-020-1145-5 32020881 PMC7001272

[157] Ali MM Akhade VS Kosalai ST Subhash S Statello L Meryet-Figuiere M. PAN-cancer analysis of S-phase enriched lncRNAs identifies oncogenic drivers and biomarkers. Nat Commun. (2018) 9:883. 10.1038/s41467-018-03265-1 29491376 PMC5830406

[158] Huarte M Guttman M Feldser D Garber M Koziol MJ Kenzelmann-Broz D. A large intergenic noncoding RNA induced by p53 mediates global gene repression in the p53 response. Cell. (2010) 142:409–19. 10.1016/j.cell.2010.06.040 20673990 PMC2956184

[159] He J Ma X. Interaction between LncRNA and UPF1 in tumors. Front Genet. (2021) 12:624905. 10.3389/fgene.2021.624905 33732285 PMC7959175

[160] El Bassit G Patel RS Carter G Shibu V Patel AA Song S. MALAT1 in human adipose stem cells modulates survival and alternative splicing of PKCδII in HT22 cells. Endocrinology. (2017) 158:183–95. 10.1210/en.2016-1819 27841943 PMC5412980

[161] Andric V Nevers A Hazra D Auxilien S Menant A Graille M. A scaffold lncRNA shapes the mitosis to meiosis switch. Nat Commun. (2021) 12:770. 10.1038/s41467-021-21032-7 33536434 PMC7859202

